# Can mothers’ representations of their infants be improved in primary care? A randomized controlled trial of a parenting intervention using video feedback in a predominantly low- to moderate-risk sample

**DOI:** 10.3389/fpsyt.2023.1232816

**Published:** 2023-09-14

**Authors:** Kjersti Sandnes, Turid Suzanne Berg-Nielsen, Stian Lydersen, Silja Berg Kårstad

**Affiliations:** Regional Centre for Child and Youth Mental Health and Child Welfare (RKBU Mid-Norway), Department of Mental Health, Faculty of Medicine and Health Sciences, Norwegian University of Science and Technology (NTNU), Trondheim, Norway

**Keywords:** maternal representations, infant, primary care, video-feedback intervention, WMCI

## Abstract

**Introduction:**

Mothers’ representations of their infants are important intervention targets because they predict the observed quality of infant–mother interactions. The current study investigated the influence of a video-feedback infant-parent intervention on mothers’ representations of their infants beyond the effect of standard treatment.

**Methods:**

Data from a naturalistic, randomized controlled trial of 152 predominantly low- to moderate-risk mothers (mean age = 29.7 years) with infants (mean age = 7.3 months) were used. At Well Baby Centers, all families followed the universal program, which was treatment as usual (TAU), whereas half of the families also received the intervention. The Working Model of the Child Interview categories and scales as well as three latent factors generated from a factor analysis were used to assess maternal representations at baseline and follow-up (9–13 months after baseline). A linear mixed model analysis was used to analyze the data.

**Results:**

There were no differences in representation changes from baseline to follow-up between the control group (TAU) and intervention group. When both groups were combined, there were minor improvements in the mothers’ representations at the follow-up.

**Discussion:**

Aspects of the intervention, the quality of TAU, and the homogeneity scores of the predominantly low-risk sample may explain the intervention’s lack of effect on mothers’ representations beyond TAU. The supportive services at Norwegian Well Baby Centers as well as the infants’ increasing age putatively contributed to the improved features of the mothers’ representations in the total sample. That standard community care may affect maternal representations has not been shown before. Future research should identify the core components in interventions targeting maternal representations and examine whether those components can be incorporated in primary care. Including measures of mothers’ reflective functioning could broaden our knowledge of representations and their changeability.

**Clinical trial registration:**

This study is registered in the International Standard Randomized Controlled Trial Number registry under the reference number ISRCTN 99793905.

## 1. Introduction

Mothers develop perceptions and expectations of their infants and of themselves as mothers already during pregnancy (1, 2). Such perceptions and expectations or representations encompass mothers’ subjective ideas, fantasies, and emotional reactions about their infant, themselves as a caregiver, and their relationship with their infant (3). Both prenatal and postnatal representations of the infant are related to observed parenting behavior, the quality of infant–mother interactions, and the infant’s development of attachment to the mother (2, 4–6). Mothers’ negative representations, which develop considerably early and significantly affect their interpretation of the infant and their behavioral responses, make them highly relevant targets for preventive, early interventions (7). Although negative representations are prevalent in both clinical and non-clinical samples, interventions aiming to improve such representations are more often tested with mothers with clinical levels of psychopathology or other risk indicators associated with a negative influence on parenting, such as low socioeconomic status, low educational level, victims of interpersonal violence, and problematic alcohol or substance use (7–9). The effectiveness of interventions suitable for mothers with lower risk are scarcely studied. Thus, the current randomized controlled trial investigated the effect of a low-threshold, video-feedback infant–parent interaction intervention, which is often used at community Well Baby Centers, on the representations of low-to-moderate risk mothers of infants.

Mothers’ representations of their infants correspond substantially with how early care experiences are currently represented in the mothers’ minds (2). A mother’s representation of her infant might emerge within the framework of her current state of mind regarding her own childhood experiences of receiving care (10). For example, a mother with a coherent and flexible representation of her past experiences of receiving care will have a greater capacity to understand her child’s signals accurately and respond sensitively to her child’s needs. Conversely, a mother who is somewhat preoccupied with past issues, losses, and trauma or is emotionally more distant and dismissive of difficult past experiences will be less able to correctly perceive the child’s signals and more likely respond insensitively (2).

Assessing representations often involves analyzing the discourse in semi-structured interviews. The Adult Attachment Interview (11) is most frequently used for assessing adults’ current state of mind regarding their own attachment experiences. For assessing mothers’ representations of their infant, two validated interviews are often used: the Working Model of the Child Interview (WMCI) (12) and Parent Development Interview (13). The current study used the WMCI, which rates the qualitative features, content, and emotional tone of a mother’s descriptions of her past and present experiences as well as future expectations of the infant (14). Her representations are classified as balanced if her discourse is coherent; her perceptions are open, flexible, and accepting of the child; and she refers to sensitive responses to the child’s needs. Alternatively, the mothers’ representations can be classified into one of the two non-balanced categories: disengaged or distorted. Disengaged representations are characterized by low emotional involvement and distant or rejecting perceptions of the child’s emotional needs, whereas distorted representations are characterized by low coherence and consistency in the mothers’ statements. Mothers with distorted representations may seem overwhelmed by their parenting responsibilities or are self-involved and distracted by other concerns (14, 15).

Using measures such as the Adult Attachment Interview, Parent Development Interview, or WMCI, researchers have reported that mothers’ representations of their infant were up to 80% stable from pregnancy until the infant was 1 year old (1, 16). Mothers’ balanced representations were more stable from pregnancy until the child was 1 year old (79%), compared to those of mothers with disengaged and distorted representations (48 and 37%, respectively) (16). Considerable stability across toddlerhood exists for mothers’ representations of their relationship with their child (17). Nonetheless, mothers’ representations can be influenced by the infant’s characteristics, other interactions parents might have, maternal psychopathology, stressful life experiences, or interventions (18, 19). For example, Theran et al. found that the representational category changed for 38% of the mothers in their sample during the infant’s first year of life (16); in the same study, a change from balanced to non-balanced representations was predicted by mothers’ depression, single parenthood, experience of interpersonal violence in pregnancy, and family income. Other maternal characteristics such as personality traits have been found to buffer or magnify the effect of interpersonal violence on maternal representations (20). Moreover, research shows that daily hassles increase anger-related aspects in mothers’ representations across toddlerhood (17), and that there are associations between children’s clinical diagnoses such as failure to thrive, sleep disorders, attention deficit hyperactivity disorder, cerebral palsy, and epilepsy, and mothers’ non-balanced representations (21–24). However, such cross-sectional studies cannot examine the direction of influence—that is, whether negative maternal representations result from or contribute to the clinical status of the infant. In one study of a non-clinical sample, mothers were more often disengaged during pregnancy than after the infant was born, which indicates that gaining experience with the child may alter mothers’ representations (25). How more specific child characteristics may influence change in mothers’ representations has not been studied extensively. One study found that premature birth influenced changes in mothers’ representations (26); however, parents’ socioeconomic status and family structure may moderate this effect (27).

To our knowledge, very few randomized controlled trials (RCTs) have documented the effect of interventions on mothers’ overall representational categories. Mom Power is an attachment-informed, group-based parenting education program; a study found that high-risk mothers who participated in the Mom Power program as an intervention had more balanced representations of their children compared to mothers who received the Mom Power curriculum through the mail (28). The Mom Power program encourages social support and self-care, guides mother-infant contact, and recommends additional care to mothers when necessary. Julian et al. introduced the Mom Power program to military families and discovered that parents (mothers and fathers) who participated in the program had more balanced representations than did parents who followed a home-based psychoeducational program (29).

Two other RCT studies have examined the effect of an intervention on the features of mothers’ representations of their infants. Suchman et al. offered the Mother and Toddler Program, a 12-week individual psychotherapy program, in addition to outpatient treatment of substance use problems for mothers of children up to 3 years of age, compared to the control condition of receiving counseling and educational pamphlets (30); the Mother and Toddler Program improved the mothers’ combined scores on measures of representational qualities such as openness, acceptance, coherence, and sensitivity.

In 2015, a systematic review concluded that parent–infant psychotherapy, which is a type of intervention designed to target parents’ representations, yielded limited evidence of improvement in representations (8). Later, Fonagy et al. reported that parent–infant psychotherapy improved aspects of helplessness and hostility in maternal representations in an adverse sample of mothers with mental health problems (31).

A concept related to parental representations is parental mentalizing, which refers to a parent’s capacity to see the child as a psychological agent with their own mental experiences and attune to the child’s mental state (32). This capacity is suggested to be the mechanism by which maternal representations influence the mother–child relationship (33), as well as the mechanism behind the effect of an intervention on maternal representations (28). The concept of parental mentalization has been operationalized to other, partly overlapping concepts such as parental reflective functioning (PRF) (32, 34, 35). The Reflective Functioning Scale was developed to measure parents’ mentalizing capacity (32). Further, PRF has a self-reflective component and a child component, which together reflect a parent’s ability to separate their own mental processes from those of the child (35). Attachment-informed interventions can improve PRF (36, 37), and prior RCTs have found that interventions may influence either self-focused or child-focused PRF (30, 36, 38, 39).

In terms of improving the infant–parent relationship in general, meta-analyses and systematic reviews conclude that interventions involving video feedback are more effective (9, 40–42). These studies included measures of parental stress, coping measures, self-confidence, and self-appreciation; however, assessments of parents’ representations of their infants were not utilized. Interventions using video feedback often focus on the interaction behavior of the mother and infant, although some programs also address the mother’s representational level by including discussions of how the mother’s past attachment experiences influence the relationship with their infant. One intervention that includes both approaches is the Video-Feedback Intervention to Promote Positive Parenting With Discussions on the Representational Level (VIPP-R) (43). Research has shown that the VIPP-R promotes maternal sensitivity and infant attachment security, but only for mothers with non-balanced representations (44). Whether the VIPP-R also alters mothers’ representations has not been reported (44). The purpose of the current study was to examine if Video Feedback of Infant–Parent Interaction (VIPI) could impact mothers’ representations of their infants as measured by the WMCI. We used data from an RCT in which VIPI improved mother–infant interaction quality compared to conventional care (22). In Norway, the VIPI intervention is frequently employed in community preventative services. Thus, investigating whether VIPI also influences representations, which presumably precede parent–infant interaction, is relevant for preventive work in primary care.

The VIPI intervention originated from the core principles of the Marte Meo method, which was developed by Maria Aarts in the Netherlands (23) as one of the first video-feedback methods to support parent-child communication and interaction and promote child development. The Marte Meo method follows a solution-focused and resource-oriented approach to working with families, and its goal is to enhance parental efficacy and sensitivity to the child’s signals and needs. Marte Meo is often used in early intervention and counseling in primary care but can also be applied to dyadic relationships across ages and contexts (45–49). It is widely implemented, and there are registered Marte Meo professionals in 39 countries across Europe, Africa, Asia, and Oceania (50).

Two RCT studies on the effect of Marte Meo have reported improved caregiver–infant interaction quality and child development outcomes (45, 51). Axberg et al. also identified a medium-to-large effect size on children’s symptoms related to antisocial behavior as reported by parents and teachers (51). Qualitative studies have reported positive effects of the Marte Meo method on maternal sensitivity and maternal depression (52, 53). According to Gill et al., Marte Meo and its focus on positive reinforcement may be able to develop and change parents’ working models of the child (30). However, this is yet to be tested quantitatively with validated measures of maternal representations.

Most Norwegian families with infants and toddlers (0–5 years) attend the universal, health-promoting, and preventive services at local Well Baby Centers (0–5 years), which offer a standard, universal health program called the Primary Child Healthcare Program (PCHP) (54, 55). The Well Baby Centers are interdisciplinary and staffed by public health nurses and doctors; however, physiotherapists, psychologists, occupational therapists, and social and educational staff are consulted when necessary. Parents receive guidance on their baby’s development and well-being, and PCHP nurses are trained to identify and support parents with mental health issues, alcohol or substance use problems, and domestic violence. Individual or group consultations are available, as is a medical examination by a doctor (31).

Although the PCHP was introduced through policy and first implemented in the 1930s, very few evaluations of its effects have been conducted. According to one study by the Norwegian School of Economics, access to Well Baby Centers had a positive effect on education and earnings, and people who attended the program as children had fewer health risks at the age of 40 years, especially those from low socioeconomic backgrounds (56).

Studies conducted in Norway and England have indicated that postpartum support from a public health nurse may reduce depression in women (57, 58). A systematic review and meta-analysis of qualitative and quantitative studies of mothers’ and fathers’ experiences during their infant’s first year also found that support from nurses at Well Baby Centers helped mothers who felt overwhelmed by being the primary caregiver (59). In particular, the opportunity to discuss and reflect upon the demands of motherhood as part of the PCHP seemed to be helpful for those mothers (59). These findings are supported by a qualitative study of Swedish first-time mothers, who reported feeling concerned and insecure during the first weeks after delivery and sought support and affirmation from nurses at child health care centers to feel more secure in their parenting role (60).

More research on the support and interventions provided at Well Baby Centers is needed. A recent policy recommendation for governments in the Nordic countries concerning infants’ first 1,000 days of life (from conception to 2 years old) identified the following areas of improvement: (I) providing comprehensive support for parents during the infant’s first 1,000 days of life; (II) identifying and responding systematically to risk factors; and (III) encouraging further research about this early period in a child’s life (61). The current study, which aimed to investigate the effect of VIPI beyond standard care, is therefore in line with these recommendations. The original RCT from which the current study drew data sought to explore the effect of VIPI on mother–infant interaction quality beyond the effect of the standard, universal PCHP provided by community Well Baby Centers (22). The study’s sample consisted of predominantly low-to-moderate risk mothers with infants (0–2 years) recruited from community Well Baby Centers. The PCHP served as the study’s control condition and was followed by all families. Furthermore, half of the families received the VIPI intervention, which had a beneficial short-term effect on the quality of mother-infant contact (22). Given that a mother’s representations predict how she interacts with her infant (3, 38), the current study considers the following research question:

Does the VIPI intervention have any effect on maternal representations compared to the effect of the standard, universal preventive care at Well Baby Centers, which is the treatment as usual (TAU) condition?

## 2. Materials and methods

We used data from a naturalistic, longitudinal, multi-site RCT that investigated the effect of the VIPI intervention on mother–infant interaction quality (45). We examined the effect of the VIPI intervention on maternal representations beyond the possible effect of the PCHP, which was the control condition (TAU). For ethical reasons, the participating families could not be randomized to no treatment or be put on a waiting list control, as that would jeopardize the infants’ health, and public health nurses cannot be instructed to treat parents differently than they should by decree.

### 2.1. Participants

The research participants were mothers of infants. During the study period, public health nurses at Well Baby Centers and other professionals recruited 180 families who were seeking help with infant–parent interaction challenges. The study took place in the cities of Oslo and Trondheim and in six rural towns in the southeastern part of Norway. The inclusion criteria were parent–child interaction problems (defined by a parent or professional) and an infant age of between 0 and 24 months. The study’s criteria for exclusion were parents with a severe mental health or developmental disorder, an ongoing substance use problem, or insufficient language skills to complete a self-report questionnaire. There were no exclusion criteria for the infants. Of the 158 eligible families, 152 enrolled and underwent a baseline evaluation. All but two participants were mothers who were on paid parental leave at the time of the study. For simplicity, the study participants are henceforth referred to as “mothers.” No incentives were offered for participation, and all participants provided written informed consent.

Table 1 presents the sample characteristics of the VIPI group (which received the VIPI intervention in addition to the PCHP), the TAU group (which received the PCHP), and the two groups combined (total sample) at the time of inclusion. For the total sample, the mean age of the participating infants was 7.3 months (SD = 5.1 months), and 48.9% of them were male. In 71.2% of the families, the included infant was the firstborn child. The mean age of the participating mothers was 29.7 years (SD = 5.6 years), and 63% of the mothers had a bachelor’s degree or higher education. Most participants were of Norwegian (79.8%) or European (6.4%) origin.

**TABLE 1:** Sample characteristics at baseline for the intervention group receiving Video-Feedback of Infant–Parent Interaction (VIPI), the group receiving treatment as usual (TAU), and the total sample (GROUP).

	VIPI (*n* = 80)			TAU (*n* = 61)			GROUP (*n* = 141)		
Baseline characteristics	*n* (%)	Mean (SD)	Range	*n* (%)	Mean (SD)	Range	*n* (%)	Mean (SD)	Range
**Child characteristics**									
Age (months)		7.8 (5.6)	1.0–20.0		6.7 (4.3)	1.3–20.0		7.3 (5.1)	1.0–20.0
Male	33 (41.8)			35 (58.3)			68 (48.9)		
First-born	53 (72.6)			41 (69.5)			94 (71.2)		
**Mother characteristics**									
Age (years)		29.4 (5.3)	19–42		30.1 (6.0)	19–43		29.7 (5.6)	19–43
**Ethnicity**									
Norwegian	41 (75.9)			34 (85.0)			75 (79.8)		
Other European	3 (5.6)			3 (7.5)			6 (6.4)		
Asian	2 (3.7)			2 (5.0)			4 (4.3)		
African	2 (3.7)			0			2 (2.1)		
South American	1 (1.9)			1 (2.5)			2 (2.1)		
**Educational level**									
High school/vocational	32 (41.0)			18 (30.0)			50 (36.2)		
Bachelors’ degree	16 (20.5)			19 (31.7)			35 (25.4)		
Masters’ degree or above	29 (37.2)			23 (38.3)			52 (37.9)		
Family income (in 1,000 N.kr)		33.96 (17.1)			33.90 (18.1)			33.9 (17.5)	

VIPI, Video Feedback of Infant–Parent Interaction intervention; TAU, treatment as usual; GROUP, total sample; SD, standard deviation; N.kr, Norwegian kroner.

In half of the families (50.9%), the parents had asked for help addressing parenting challenges. For the rest of the families, participation in the study was voluntary but recommended by public health nurses or other professionals. The most frequently cited reasons for participating were as follows: concern regarding the infant’s regulation (32.6%), parent–child interactional challenges (14.5%), wanting to learn more (10.8%), parent’s mental health (3.6%), developmental delay of the child (3.2%), social development of the child (2.4%), and a need for support (2.2%). Altogether, these reasons were stated by 69.3% of the participants. As their reasons for recruiting, the professionals reported subjective estimations of maternal depressive symptoms (60–70%), interest in parenting (10–20%), concern about the child’s development (10%), and insensitive parenting (10%).

Five families received support (financial or otherwise) from the Norwegian Child Welfare Services. Although parent–infant interaction problems were a criterion for inclusion, prior investigations using the same data as the current study, reported that there were no risk indicators associated with negative effects on parenting among the mothers (depression, anxiety, stress, alcohol use, income, and level of education), infants (developmental status), or in terms of the mean quality of infant–mother interaction (45, 62). The sample in this study was relatively homogeneous. Most participants were considered predominantly low-to-moderate risk, and only a few were high-risk cases. Thus, we did not expect any confounding effect of the sample’s risk factors.

### 2.2. Procedure

Data were collected at baseline (*N* = 152) and at follow-up 6 months after the end of the VIPI intervention (*n* = 112). The intervention period lasted 3 months, and the total study period lasted 9–13 months (*M* = 11.3 months). Three trained research assistants with bachelor’s degrees in preschool education, nursing, or social work visited the participants’ homes twice or thrice over the course of 1 to 2 weeks to collect data. A demographic interview was conducted at both baseline and follow-up, and our main outcome variable (WMCI scores) was assessed only at these two time points for two reasons. The first was to minimize assessment burden for the participating mothers, as the WMCI takes up to 90 min to complete. The second was to avoid having the mothers participate in the second interview shortly after the first, and thus, repeat their responses. The WMCI was conducted for mothers a few days after the initial visit (40). To aid coding, the interview was filmed. Ten interviews were lost or omitted from the data because of poor recording quality. Consequently, 142 baseline interviews were successfully coded, and after accounting for the missed or excluded interviews, 104 interviews were coded at the follow-up.

### 2.3. Primary Child Healthcare Program

All participating families followed the PCHP provided by community Well Baby Centers. Although the frequency of routine health visits varied slightly between the included Well Baby Centers, all families had a minimum of one home visit from a midwife within 1 or 2 weeks after delivery, and they visited their local Well Baby Center at 6 weeks and 3, 4, 6, 8, 10, 12, 15, 18, and 24 months. The visits were conducted in either an individual or group setting, and they included pediatric check-ups when the infants were 3, 12, and 24 months old. All families in both groups could seek help, support, and advice from other professionals; however, these professionals were instructed not to conduct any video-feedback intervention.

### 2.4. Randomization

**FIGURE 1:**
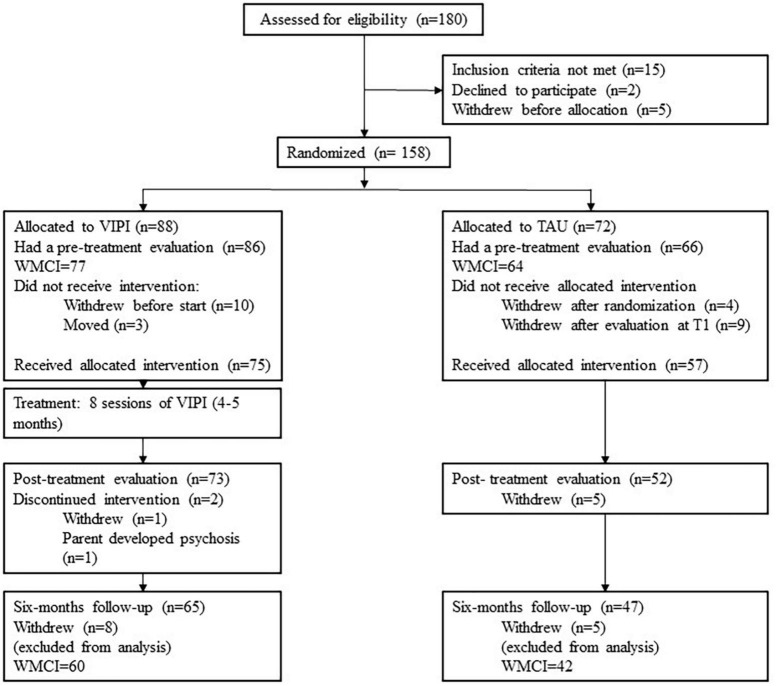
Flowchart of the recruitment and randomization processes.

Figure 1 depicts a flowchart of the recruitment and randomization processes. After the baseline assessment, the participants were randomized to one of two groups—VIPI or TAU—by a successive 1-2-1-2 allocation ratio within each urban district or rural municipality. The inequality of the group sizes (VIPI: *n* = 88, TAU: *n* = 72) could have been due to the allocation starting and ending with the same number (1 = VIPI). Additionally, five pairs of siblings or twins were allocated to the same treatment group, which influenced group size. The research assistants were blinded to the randomization status of the families from whom they collected and handled data.

The cumulative drop-out rates from baseline to 6 months follow-up after intervention were 26.2% for the VIPI group and 34.6% for the TAU group, which is reasonable given that participation in the study required 9–13 months. The majority dropped out from the study after being assigned to the VIPI or TAU group. Table 2 shows the baseline descriptive statistics for the mothers and children’s age, WMCI factors, and WMCI scales for the main sample and the attrition group across the VIPI and TAU groups.

**TABLE 2:** Descriptive statistics for the main sample and attrition group, for the group receiving Video-Feedback of Infant–Parent Interaction (VIPI) intervention and the group receiving treatment as usual (TAU) separately.

	Main sample				Attrition group			
	VIPI		TAU		VIPI		TAU	
	*n*	Mean (SD)	*n*	Mean (SD)	*n*	Mean (SD)	*n*	Mean (SD)
**Variables**								
Child age (months)	80	7.8 (5.6)	61	6.6 (4.3)	17	7.4 (5.9)	16	9.3 (5.1)
Mother’s age (years)	80	29.4 (5.3)	61	31.1 (6.0)	17	26.9 (5.5)	16	28.9 (6.9)
**WMCI variables**								
Factor 1 balanced	76	3.31 (0.85)	64	3.25 (0.93)	17	3.20 (0.80)	16	2.74 (0.74)
Factor 2 resentful	76	1.71 (0.67)	64	1.67 (0.73)	17	1.78 (0.75)	16	1.95 (0.83)
Factor 3 apprehensive	76	1.82 (0.87)	64	1.82 (0.99)	17	1.87 (1.03)	16	2.06 (1.14)
Richness of perception	76	5.40 (0.99)	64	3.30 (1.06)	17	3.09 (1.00)	16	2.69 (0.70)
Openness to change	76	3.47 (0.95)	64	3.32 (0.99)	17	3.32 (0.95)	16	2.81 (0.89)
Intensity of involvement	76	3.47 (0.95)	64	3.32 (0.99)	17	3.32 (0.95)	16	2.81 (0.89)
Coherence	73	3.32 (1.04)	64	3.16 (1.08)	17	3.41 (0.96)	16	2.56 (0.73)
Sensitivity	73	3.32 (0.97)	64	3.47 (0.93)	17	3.23 (0.75)	16	2.91 (0.86)
Acceptance	75	3.42 (0.92)	64	3.48 (0.93)	17	3.32 (0.81)	16	3.06 (0.77)
Joy	76	3.07 (0.92)	64	3.07 (0.98)	17	3.09 (0.89)	16	3.06 (0.77)
Pride	76	2.91 (0.94)	64	2.87 (1.09)	17	2.77 (0.77)	16	2.42 (0.91)
Anger	76	1.67 (0.82)	64	1.59 (0.84)	17	1.79 (1.08)	16	1.88 (0.96)
Disappointment	76	1.36 (0.67)	64	1.38 (0.62)	17	1.44 (0.86)	16	1.59 (0.80)
Anxiety	76	1.80 (1.00)	64	1.81 (1.06)	17	1.77 (1.09)	16	2.03 (1.22)
Guilt	76	1.31 (0.65)	64	1.38 (0.70)	17	1.15 (0.49)	16	1.50 (0.82)
Indifference	76	1.56 (0.82)	64	1.43 (0.67)	17	1.82 (1.02)	16	1.63 (0.74)
Child difficulty	76	2.11 (0.96)	64	2.06 (1.04)	17	2.12 (0.94)	16	2.38 (1.03)
Fear for infant’s safety	76	1.85 (0.88)	64	1.82 (1.01)	17	1.97 (1.07)	16	2.10 (1.10)

VIPI, Video Feedback of Infant–Parent Interaction intervention; TAU, treatment as usual; WMCI, Working Model of the Child Interview; SD, standard deviation.

### 2.5. Video Feedback of Infant–Parent Interaction intervention

To standardize the VIPI intervention, three experienced Marte Meo supervisors developed the VIPI manual, which describes intervention steps for families with infants aged 0–2 years. The VIPI intervention is limited to eight weekly sessions, but the last two can be tailored to the individual needs of the family. The duration of each session is not pre-defined; however, it usually lasts about 1 h. Mandatory homework is assigned between sessions. Further, parents are asked to register moments of interaction with their infants in relation to new topics introduced during the feedback session, for which they are given a registration form.

Eight experienced and certified Marte Meo therapists completed a 2-day training on using the VIPI manual, followed by supervision with a licensed Marte Meo supervisor. This supervision included discussions of the videotapes of infant–mother interaction and the therapists’ feedback to the mothers. To ensure treatment fidelity, the supervisor reviewed the videotapes of the therapists’ feedback to the mothers, how they used the registration forms provided by the manual, and how they explained the core elements of the intervention. None of the therapists deviated from the manual’s instructions.

The families in the intervention group completed six to eight at-home video-feedback sessions with the VIPI therapist. The therapist used a selection of pre-recorded video clips of parent–infant interactions that demonstrated the following core elements of the Marte Meo method: identifying the infant’s initiatives; attunement and timing of the parent’s responses to the infant’s initiatives; following the child to support synchronicity; naming the infant’s initiative, emotions, actions, relational activities, and transitional situations; structured step-by-step guidance when interacting; and directing attention toward social interaction and exploration. The pace at which new elements were introduced was dependent on how the parents responded to the intervention. In some families where both parents received the VIPI, the therapist gave individual feedback based on a separate videotape for each parent–infant dyad. For these families, data from only one of the parents were assessed and included in the analyses. While reviewing the video clips, sensitive parenting practices were reinforced through a reflective dialogue between the therapist and parent to build an understanding of the child’s state of mind and scaffold parenting practices in daily routine situations.

### 2.6. Measures

#### 2.6.1. Demographics

The research assistants interviewed all participants to collect demographic and socio-economic information.

#### 2.6.2. Working Model of the Child Interview

The WMCI was conducted for all mothers at baseline and a follow-up occurring 9–13 months after the baseline. It is a validated interview for eliciting parents’ perceptions, emotions, expectations, and cognitions of their specific child, their relationship with their child, and their own role as a caregiver. The WMCI includes questions about the pregnancy, present, and future (15), which typically concern situations where the child’s attachment needs are activated (e.g., the child is ill, frightened, or hurt). The questions, for example, include, “What about your child’s behavior now is most difficult for you to handle? Can you give me a typical example? What do you feel like doing when your child reacts this way? How do you feel when your child reacts this way? What do you actually do?” (14). The WMCI categories have adequate validity and reliability (5). The stability of these categories is the strongest for balanced representations, and the WMCI qualitative scales are more stable than the two content scales (1).

The interviews were video recorded to facilitate coding. Each mother’s descriptions and affective tone throughout the interview were rated on 15 five-point Likert scales (1 = none; 5 = extreme). Six of these scales address six respective qualitative features of the representation. The first is the richness of perception, which conveys the level of detail in the mother’s descriptions of the child and how well the mother knows the child. The second, openness to change, refers to accommodating new information about the child and their changing developmental needs. The third, the intensity of involvement, is the degree of emotional immersion in and preoccupation with the child. The fourth, coherence, signifies the clarity, organization, and consistency of the descriptions. The fifth, caregiving sensitivity, is the ability to understand the child’s experiences and respond to their needs accordingly. The sixth and last qualitative feature is acceptance, which describes the extent to which the mother accepts the child as they are and accepts the responsibilities of being a mother. The next two scales relate to the specific content themes of infant difficulty (i.e., perceiving the infant as difficult to relate to and care for) and fear for infant safety (i.e., irrationally worrying about the infant’s safety or death of the child). The last seven scales measure the presence of seven respective affective tones during the interview: joy, pride, anxiety, anger, guilt, indifference, and disappointment.

Based on the patterns of the scale ratings, representations were assigned to one of three global categories: balanced, disengaged, or distorted. A two-way categorization of representations into balanced and non-balanced, wherein the disengaged and distorted categories are collapsed within the non-balanced category, could also be applied. A balanced parental representation is characterized by warm, coherent, and elaborate descriptions of the child that integrate the positive and challenging aspects of the child’s personality as well as the relationship between the caregiver and child. Such representations indicate not being overwhelmed by the child’s needs; further, they denote the acceptance and acknowledgment of the child’s individuality and subjective experience and the perception of the relationship as valuable for both the parent and child.

A disengaged parental representation reflects an emotionally distant, cognitive, or intellectual approach to the child and parenting. It describes the child in a general and less affectively involved manner. This type of representation can also signify an elevated level of indifference, rejection of the child’s needs, and sometimes even an aversion to the child.

A distorted parental representation is illustrated by inconsistent, incoherent, and contradictory descriptions of the child and the parent’s relationship with the child. Parents with representations that fall in this category may be self-involved or preoccupied with other matters. Sometimes, they are unsure or anxiously overwhelmed by the child’s needs and are unable to remain focused on the child.

Two certified raters coded the WMCI interviews: one has a master’s degree in preschool education and the other is a clinical psychologist. The raters were blinded to the randomization status of the families but not to the times of the interviews. There are very few reliable WMCI raters in Scandinavia. At the beginning of this project, we did not know if we would be able to find reliable raters to code all the interviews from both time points. Thus, only the pre-intervention interviews were coded at first. About 6 months later, the same raters agreed to code the WMCI data from the 6-month follow-up. All interviews were coded from recordings; consequently, we could not prevent the raters from being influenced by their first-time ratings.

At pre-intervention, the first rater coded 108 interviews and the second rater coded 55 interviews; 20 of these interviews were double-coded. At the 6-month follow-up, the first rater coded 104 interviews and the second rater coded 18 interviews; 18 of these interviews were double-coded. The interrater reliability of the WMCI clinical scales was good for five scales, moderate for nine, and fair for one (Cohen’s weighted κ mean = 0.539) (63). A relatively low variance in some scales may have contributed to some of the low kappa values for the WMCI scales (64). The value of Cohen’s κ was 0.898 [95% confidence interval (CI): 0.704–1.00] for the balanced, disengaged, and distorted categories and 0.886 (CI: 0.671–1.00) for the balanced versus non-balanced (i.e., disengaged, and distorted) categories. Therefore, we concluded that the interrater agreement was acceptable and the 15 clinical scales would be used in further analyses (62). For the current analyses, we used both the WMCI categories and the 15 clinical scales, as global categories of representations might not be sufficiently sensitive to capture subtle yet clinically significant changes (65). In addition, we adopted three WMCI factors that were derived from an examination of the psychometric properties of the WMCI in this sample. A factor analysis yielded three factors denoted by “balanced,” “resentful,” and “apprehensive.” Factor 1, “balanced,” corresponded to the original balanced category and was loaded by the WMCI clinical scales of richness of perception, openness to change, intensity of involvement, coherence, acceptance, sensitivity, joy, and pride. Factor 2, “resentful,” was loaded by the WMCI clinical scales of anger, disappointment, and child difficulty. Factor 3, “apprehensive,” was loaded by the WMCI scales of anxiety and fear for infant safety. Both factor 2 “resentful” and factor 3 “apprehensive” corresponded to the original non-balanced categories. The three derived factors showed evidence of factorial, concurrent, and discriminant validity (62).

### 2.7. Statistical analyses

We conducted a power analysis prior to the study. For an expected standardized difference of 0.5 between the VIPI and TAU groups, 60 families in each group were required for a power of 78% at a 5% significance level (22).

We conducted linear mixed model analyses with the three WMCI factors (balanced, resentful, and apprehensive) and the 15 WMCI clinical scales as dependent variables one at a time. Time (follow-up versus baseline) and the interaction between time and intervention (VIPI versus TAU) were included as fixed factors to determine whether the change was different between the two groups. We adjusted for the baseline value of the dependent variable as recommended by Twisk et al. with equation (2c) (44). Participants were included as a random effect.

We investigated change in the WMCI categories (balanced, disengaged, and distorted) from baseline to follow-up for the VIPI group, TAU group, and total sample separately. The change from baseline to follow-up was analyzed separately for each group and the total sample using the Stuart-Maxwell test of marginal homogeneity.

We found no differences between the groups in terms of change in the WMCI factors and scales. However, changes were observed in the mothers’ representations in both groups. Therefore, we conducted additional linear mixed model analyses with time as a fixed factor (follow-up versus baseline) to investigate changes in the WMCI factors and clinical scales for the total sample. Finally, we added the child’s age at follow-up to investigate whether any changes in maternal representations could be explained by the fact that the child had grown older.

Significant differences between the groups at baseline were not tested, as recommended by Dumville et al. (66), Fayers and King (67), Lydersen (68), Lydersen (69), De Boer et al. (70). It may seem that missing data depend on baseline values (see Table 2). This is appropriately handled in linear mixed model analysis, such that the results are unbiased under this type of deviation from missing completely at random.

We estimated the WMCI factor scores as the mean of available scores on the scales if data were available for at least half of the scales. Otherwise, we handled missing values by available case analysis such that, in each analysis, we included the observations with complete data for the relevant variables. A linear mixed model includes all participants with data from at least one-time point. The results are unbiased if data are missing at random, whereas a complete case analysis including only participants with data from both time points would be unbiased only under the more restrictive missing completely at random assumption. We regarded *p*-values of less than 0.05 as statistically significant; however, because of multiple hypotheses, we interpreted *p*-values between 0.05 and 0.01 with caution. We report 95% CIs where relevant. All analyses were conducted using SPSS 27.

### 2.8. Ethics

We used data from an original study titled “Video feedback compared to TAU in families with parent–child interactions problems: a randomized controlled trial” (45). The collection and storage of data for the original study were approved by the Norwegian Centre for Research Data and later by the Regional Committee for Research Ethics in Mid-Norway (REC; reference number 1.2007.2176). The study is registered in the International Standard Randomized Controlled Trial Number registry under the reference number ISRCTN 99793905. All participants provided written consent. Our study was found to be exempt (REC reference 2017/1723) because the data from the original study had been anonymized.

## 3. Results

Table 3 presents descriptive statistics for the WMCI measures at baseline and follow-up and the results for estimated treatment effects from the linear mixed model analyses. There were no statistically significant differences in change from baseline to follow-up between mothers in the VIPI and TAU groups for any of the three WMCI factors or fifteen clinical scales.

**TABLE 3:** Descriptive statistics for the Working Model of the Child Interview (WMCI) measures at baseline and follow-up, and estimated treatment effect of the Video Feedback of Infant–Parent Interaction intervention (VIPI) vs. treatment as usual (TAU) from mixed model analyses.

	Intervention (VIPI) (*n* = 76)			Control (TAU) (*n* = 64)			Difference (group × time)		
Variablesn	*n*	Mean	SD	*n*	Mean	SD	Estimate	95% CI	*p*-Value
**Factor 1 balanced**									
Baseline	76	3.31	0.85	64	3.25	0.93			
Follow-up	61	3.13	0.63	42	3.11	0.70	0.06	−0.25 to 0.36	0.71
**Factor 2 resentful**									
Baseline	76	1.71	0.67	64	1.67	0.73			
Follow-up	61	1.64	0.65	42	1.48	0.50	0.11	−0.13 to 0.36	0.37
**Factor 3 apprehensive**									
Baseline	76	1.82	0.87	64	1.82	0.99			
Follow-up	61	1.41	0.50	42	1.33	0.53	0.01	−0.28 to 0.30	0.95
**Perceptions**									
Baseline	76	3.40	0.99	64	3.30	1.06			
Follow-up	61	3.20	0.79	42	3.24	0.81	−0.01	−0.35 to 0.34	0.97
**Openness**									
Baseline	76	3.47	0.95	64	3.32	0.99			
Follow-up	61	3.23	0.80	42	3.18	0.72	0.06	−0.28 to 0.39	0.75
**Involvement**									
Baseline	76	3.47	0.95	64	3.32	0.99			
Follow-up	61	3.23	0.65	42	3.18	0.75	0.05	−0.28 to 0.38	0.76
**Coherence**									
Baseline	73	3.32	1.04	64	3.16	1.08			
Follow-up	60	3.12	0.76	41	3.02	0.81	0.12	−0.25 to 0.49	0.51
**Sensitivity**									
Baseline	73	3.36	0.97	64	3.47	0.97			
Follow-up	61	3.16	0.69	42	3.10	0.72	0.15	−0.18 to 0.48	0.37
**Acceptance**									
Baseline	75	3.42	0.92	64	3.48	0.93			
Follow-up	61	3.30	0.77	42	3.21	0.84	0.10	−0.24 to 0.44	0.57
**Joy**									
Baseline	76	3.07	0.92	64	3.07	0.98			
Follow-up	61	2.96	0.65	42	3.06	0.81	−0.06	−0.39 to 0.27	0.73
**Pride**									
Baseline	76	2.91	0.94	64	2.87	1.08			
Follow-up	61	2.89	0.70	41	2.87	0.76	0.03	−0.31 to 0.38	0.85
**Anger**									
Baseline	76	1.67	0.82	64	1.59	0.84			
Follow-up	61	1.66	0.76	42	1.42	0.58	0.19	−0.11 to 0.48	0.22
**Disappointment**									
Baseline	76	1.36	0.67	64	1.38	0.62			
Follow-up	61	1.36	0.61	42	1.29	0.51	0.06	−0.18 to 0.30	0.60
**Anxiety**									
Baseline	76	1.80	1.00	64	1.81	1.06			
Follow-up	61	1.42	0.57	42	1.36	0.82	0.01	−0.33 to 0.34	0.97
**Guilt**									
Baseline	76	1.31	0.65	64	1.38	0.70			
Follow-up	61	1.36	0.62	42	1.21	0.47	0.19	−0.05 to 0.42	0.12
**Indifference**									
Baseline	76	1.56	0.82	64	1.43	0.67			
Follow-up	61	1.46	0.57	42	1.55	0.73	−0.15	−0.41 to 0.11	0.26
**Child difficulty**									
Baseline	76	2.11	0.96	64	2.06	1.04			
Follow-up	61	1.90	0.78	42	1.73	0.74	0.11	−0.22 to 0.45	0.52
**Fear for infant’s safety**									
Baseline	76	1.85	0.88	64	1.82	1.01			
Follow-up	61	1.39	0.55	42	1.31	0.51	0.06	−0.25 to 0.37	0.70

WMCI, Working Model of the Child Interview; VIPI, Video Feedback of Infant–Parent Interaction intervention; TAU, treatment as usual; SD, standard deviation; CI 95%, confidence interval.

Changes in the WMCI categories at baseline and at follow-up for the VIPI group, TAU group, and total sample are presented in Table 4. The changes were not significant in the VIPI group, TAU group, or total sample (*p*-values for the Stuart-Maxwell test were 0.50, 0.22, and 0.16, respectively).

**TABLE 4:** Change in the Working Model of the Child Interview (WMCI) categories from baseline to 6-month follow-up for the intervention group receiving Video-Feedback of the Infant–Parent Interaction (VIPI), the control group receiving treatment as usual (TAU), and total sample (Total).

Group	WMCI category at baseline	WMCI category at follow-up				
		Balanced	Disengaged	Distorted	Total	Missing
TAU	Balanced	22	3	0	25	12
	Disengaged	4	2	1	7	7
	Distorted	2	3	2	7	6
	Total	28	8	3	39	26
	Missing	0	1	1	3	1
VIPI	Balanced	23	3	4	30	12
	Disengaged	5	3	0	8	9
	Distorted	6	2	2	10	3
	Total	34	8	6	48	24
	Missing	6	2	4	12	1
Total	Balanced	45	6	4	55	24
	Disengaged	9	5	1	15	16
	Distorted	8	5	4	17	9
	Total	62	16	9	87	49
	Missing	6	3	5	14	2

WMCI, Working Model of the Child Interview; TAU, treatment as usual; VIPI, Video Feedback of Infant–Parent Interaction intervention.

We observed changes in the WMCI factors and scales from baseline to follow-up for the total sample. For primary care service providers, it is clinically relevant to know whether their practices contribute to improving mothers’ representations, which predict the quality of the infant-mother relationship. Therefore, we conducted additional analyses to further investigate these changes. Table 5 shows results from the mixed model analyses of change from baseline to follow-up, both unadjusted and adjusted for the child’s age. For the total sample, we found that the estimates of Factor 3 “apprehensive” were reduced from baseline to follow-up. Furthermore, at follow-up, the mothers’ scores on the WMCI clinical scales for sensitivity, anxiety, infant difficulty, and fear for infant safety decreased from the baseline values. The child’s age at follow-up had no notable effect on the estimated mean scores at follow-up for Factor 3 “apprehensive” and the WMCI scales of anxiety, child difficulty, and fear for infant safety. However, the fact that the child had grown older, to a large extent, accounted for the reduced scores for the WMCI scale of sensitivity. For Factor 2 “resentful” and the WMCI scale of anger, we found a significant reduction of the estimated scores from baseline to follow-up only when we adjusted for the child’s age at follow-up, which is possibly a suppression effect (71).

**TABLE 5:** Results from the mixed model analyses with estimates of change from baseline to follow-up for the total sample, unadjusted and adjusted for age of child, with the Working Model of the Child Interview (WMCI) measures as dependent variables.

	Unadjusted for age			Adjusted for age		
Variables	Estimate	95% CI	*p*-Value	Estimate	95% CI	*p*-Value
Factor 1 balanced	−0.18	−0.36 to −0.01	0.049	−0.07	−0.35 to 0.21	0.626
Factor 2 resentful	−0.11	−0.25 to 0.03	0.128	−0.38	**−0.61 to −0.14**	**0.002**
Factor 3 apprehensive	−0.43	**−0.60 to −0.26**	**<0.001**	−0.50	**−0.78 to −0.22**	**<0.001**
Perceptions	−0.18	−0.38 to 0.01	0.068	−0.09	−0.43 to 0.24	0.582
Openness	−0.21	−0.40 to −0.01	0.040	0.00	−0.31 to 0.31	0.998
Involvement	−0.20	−0.40 to −0.01	0.044	−0.08	−0.39 to 0.22	0.597
Coherence	−0.20	−0.42 to 0.02	0.078	0.01	−0.34 to 0.35	0.988
Sensitivity	−0.29	**−0.49 to −0.09**	**0.005**	−0.11	−0.42 to 0.21	0.499
Acceptance	−0.20	−0.40 to 0.01	0.065	−0.07	−0.38 to 0.24	0.653
Joy	−0.08	−0.28 to 0.11	0.406	−0.03	−0.35 to 0.28	0.835
Pride	−0.03	−0.23 to 0.17	0.773	−0.05	−0.37 to 0.27	0.745
Anger	−0.06	−0.23 to 0.11	0.517	−0.37	**−0.64 to −0.09**	**0.009**
Disappointment	−0.04	−0.19 to 0.12	0.650	−0.21	−0.44 to 0.02	0.069
Anxiety	−0.41	**−0.60 to −0.21**	**<0.001**	−0.52	**−0.84 to −0.19**	**0.002**
Guilt	−0.04	−0.18 to 0.10	0.574	−0.24	−0.47 to −0.01	0.042
Indifference	0.004	−0.15 to 0.15	0.960	−0.14	−0.40 to 0.11	0.269
Child difficulty	−0.25	**−0.44 to −0.05**	**0.012**	−0.56	**−0.89 to 0.24**	**<0.001**
Fear for infant’s safety	−0.47	**−0.67 to −0.28**	**<0.001**	−0.47	**−0.76 to −0.19**	**0.001**

WMCI, Working Model of the Child Interview; CI 95%, confidence interval. Bold text signifies *p* < 0.01.

We also observed a slight decrease in the estimated mean scores for Factor 1 “balanced” and the WMCI clinical scales of openness to change and intensity of involvement. However, the *p*-values ranged between 0.01 and 0.05, and therefore, were interpreted with caution.

## 4. Discussion

To our knowledge, this is the first naturalistic, multi-site, RCT to investigate whether a video-feedback mother–infant interaction intervention (VIPI) can improve features of mothers’ representations of their infants in a predominantly low-to-moderate risk sample. Between baseline and follow-up (6 months after the intervention), we found no evidence of the effect of the VIPI on the mothers’ representations beyond TAU. For the total sample, we observed a small decrease in the features of mothers’ representations measured by some of the WMCI factors and clinical scales.

First, it is possible that the VIPI intervention in this study did not target maternal representations sufficiently to alter them. Some scholars have proposed that a key component for changing the aspects of maternal representations is to invite the mother to reflect on her own early attachment experiences and how those influence the present relationship with the child (7, 30, 36, 38). However, in the VIPI intervention, the focus of the reflective dialogue between the therapist and parent when reviewing the video clips was mainly to understand the child’s state of mind and scaffold sensitive parenting practices in daily routines. The intervention was not specifically designed to explore and elaborate upon the origins of the parent’s perceptions. Putatively, the VIPI intervention may be insufficient to improve the aspects of maternal representations measured by the WMCI. However, it is possible that the VIPI influenced the mothers’ ability to mentalize their infant, as the intervention stimulates mothers to reflect on the infant’s mental processes. Prior studies have shown that video-feedback interventions can affect mothers’ reflective functioning (36, 38). Two RCTs of attachment-informed interventions that demonstrated evidence of effects on the representations of high-risk mothers also improved their reflective functioning (28, 30). However, parent–infant psychotherapy, which addresses mothers’ mentalizing ability, did not alter mothers’ reflective functioning in a previous study, although the qualities of their representations changed (31). Despite inconsistent results from past studies, administering the Reflective Functioning Scale together with the WMCI in the current sample may have helped detect relevant changes and should be considered in future research.

Second, the universal, preventive Primary Child Healthcare Program (PCHP) at community Well Baby Centers provides extensive, high-quality services from birth through toddlerhood, which could have masked any possible effect of the VIPI intervention. The PCHP includes home visits, individual and group-based consultations, and access to various specialists if needed. Other studies have reported that support from public health nurses at the community PCHP helped first-time mothers feel secure and develop in their parenting role (60), and reduced symptoms of postpartum depression in mothers (57, 58). Similar results were found in a Swedish study (72) that examined a sample comparable to the present sample. In the Swedish study, no differences were found in the WMCI categories between a group of mothers receiving parent–infant psychotherapy and a group following the standard healthcare program at local child healthcare centers (72).

Third, it is also possible that the lack of an effect of the VIPI intervention may be because of the sample, which was predominantly low-to-moderate risk. The mean scores of the WMCI factors and scales indicated no risk, meaning that for these mothers, there was little room for improvement in their representations and a ceiling effect may have occurred. Earlier studies reporting an effect of interventions on maternal representations were conducted with heterogeneous high-risk samples with clearly negative representations, for which improvements are more likely to be detected (28–31, 73, 74). Relatedly, the mothers who dropped-out from TAU were older, less balanced, more resentful, and more apprehensive than mothers who dropped out from VIPI (Table 2). Also, their infants were older than the infants of the mothers in the drop-out group from VIPI. Possibly, the differences between the VIPI-group and the TAU-group were not unbiased. However, this potential bias should be of little concern since the linear mixed model analysis handles this in a way that makes the results unbiased under this type of deviation from missing completely at random.

The VIPI intervention had no effect on the mothers’ representations at follow-up beyond that of the TAU. Nevertheless, we observed that some aspects of the representations improved in both groups, which could imply that the primary care services contributed to the mothers’ improved representations. Research on how a universal program influences mothers with infants is scarce; thus, this finding is relevant and in line with policy recommendations (61). We conducted additional analyses that were not part of the original study, and observed minor yet significant reductions in anxiety, fear for infant safety, and assessment of the child as difficult to care for at follow-up compared to baseline for the entire sample. Nearly three-quarters of the sample was comprised of first-time mothers, who probably felt uneasy about motherhood and caring for an infant. The support, guidance, and knowledge provided by the public health nurses at Well Baby Centers may have made the mothers feel less anxious and fearful and encouraged perceptions of their child as easier to care for. To the best of our knowledge, no other RCTs have found an effect of a standard, preventive, and low-threshold community care program on negative maternal representations. The present finding may inspire an emphasis on high-quality primary care services for infants, which may be capable of influencing fundamental psychological processes in the mothers.

Our results also confirm that mothers’ representations may adjust to their child’s characteristics, such as the increasing age of the child. In the present sample, the mothers’ representations became slightly less sensitive as the child became older. An earlier study reported that high-risk mothers were less sensitive to the demands of an increasingly autonomous toddler compared to that of an infant (75). Our results confirm the same tendency in a low-to-moderate risk sample. Interestingly, the age of the child had a suppression effect on change from baseline to follow-up on the factor “resentful” and the WMCI scale of anger. A suppression effect is a negative confounding effect, meaning that adding a particular variable to the regression equation increases the magnitude of the relation between the independent and dependent variables (71). In our study, this means that the mothers in both groups reduced their representational anger and resentment, most likely because of the supportive services at the Well Baby Centers, but this effect was practically canceled out by the anger and resentment that increased as the child grew older. These findings indicate that the features of the mothers’ representations may be adjusted by child characteristics, such as the age of the child.

### 4.1. Strengths and limitations

A major strength of our study is its RCT design with two assessment points using the WMCI categories, fifteen clinical WMCI scales, and three factors that were derived from a factor analysis. The design allowed for analyses of change in maternal representations measured both categorically and continuously. The latter approach is found to be more sensitive for detecting change than when measuring categorically (30). In addition, the naturalistic quality of the study is an advantage. All data were collected at the participants’ homes; being in a familiar setting increases the likelihood that the mothers and infants were comfortable, which would strengthen the external validity of the WMCI interviews.

Nevertheless, the study has several limitations. First, although fathers were invited to participate in the study, only two participated in the interviews. Therefore, the sample consisted almost exclusively of mothers. Previous research has determined that fathers’ and mothers’ representations of their children differ (76). Thus, our results cannot be generalized to fathers. Future studies should include fathers to investigate changes across time in paternal representations as measured by the continuous WMCI scales.

Second, the naturalistic design complicates the research despite its benefits. Without a group that did not attend the PCHP, we cannot conclude that the changes in the features of the mothers’ representations of their infants were due to the PCHP. The participants were recruited by professionals who were bound by guidelines and ethical standards. Hence, the research team could apply only a few restrictions to the services at the Well Baby Centers. Furthermore, all families had to be given the option to seek help from other professionals, if necessary, which could have interfered with the effect of the VIPI intervention.

Third, the WMCI was conducted first at baseline and subsequently 6 months after the VIPI intervention. Thus, we do not know if VIPI in addition to the PCHP influenced the mothers’ representations immediately after the VIPI intervention ended. Moreover, the lack of post-intervention measures of the mothers’ representations made it difficult to control for variables that could have influenced the results. Although we controlled for time and the child’s age at follow-up, other unknown variables could have played a role as well.

Fourth, by today’s standards, the randomization procedure of a sequential 1-2-1-2 allocation is not ideal. The study was designed, and enrollment began before 2010, when the Consort Guidelines were effectuated. We cannot rule out the possibility that the recruiters were aware of the allocation pattern, which may have influenced their decision to recruit or not recruit certain families. Some of the nurses at the Well Baby Centers were hesitant to recruit families because they did not want to risk families in need being allocated to the TAU group.

The fifth limitation pertains to the WMCI raters, who were not blinded to the time of the interview. As very few reliable WMCI raters were available, the same raters coded the WMCIs from both time points. Further, all WMCIs were coded from video recordings; thus, they were not deidentified. However, the raters were blinded to whether the participants had received the VIPI intervention or only attended the PCHP at the Well Baby Centers. Therefore, this information should not have influenced their ratings.

Finally, analyzing the WMCIs for improvements in the mothers’ reflective functioning could have identified an effect of the VIPI intervention compared to TAU. Future research should consider administering the Reflective Functioning Scale along with the WMCI when investigating effects of the VIPI.

## 5. Conclusion

The present study did not find evidence of an effect of the VIPI intervention among predominantly low-to-moderate-risk mothers’ representations of their infant, possibly because the mothers’ own attachment history or state of mind in attachment-related situations was not addressed. The present findings validated prior studies’ findings regarding the relative stability of mothers’ representations of their infants. Notably, additional analyses of all participants revealed that mothers changed several aspects of their representations of their infant over a period of 9–13 months. The supportive and promotive services provided by community Well Baby Centers may have contributed to the improvement in the anxious and negative features of first-time mothers’ representations. Additionally, the aspects of mothers’ representations change with the increasing age of their infants and toddlers, which is in line with previous findings indicating that although maternal representations are stable, some features may change in response to support and the child’s developmental changes (17, 28, 30, 31). The WMCI clinical scales and variables are suggested for use in research and clinical practice because they are sensitive in evaluating subtle yet clinically relevant changes. Future research is required to investigate the efficacy of representation-targeted treatments tailored to and available in primary care services. Assessing mothers’ reflective functioning might provide more detailed insights regarding changes in maternal representations of infants.

## Data availability statement

The raw data supporting the conclusions of this article will be made available by the authors, without undue reservation.

## Ethics statement

The studies involving humans were approved by the Regional Committee for Research Ethics in Mid-Norway (REC). The studies were conducted in accordance with the local legislation and institutional requirements. Written informed consent for participation in this study was provided by the participants’ legal guardians/next of kin.

## Author contributions

TB-N and SK contributed equally to the conception and design of the study. SL contributed to the statistical analyses. KS wrote the first draft of the manuscript. All authors contributed to manuscript revision, read, and approved the submitted version.
